# Increasing sensitivity—a common-sense approach?

**DOI:** 10.1007/s12471-019-1280-z

**Published:** 2019-05-02

**Authors:** M. Michels, F. W. Asselbergs, J. van der Velden

**Affiliations:** 1 Department of Cardiology, Erasmus Medical Center Rotterdam, Rotterdam, The Netherlands; 2 Department of Cardiology, University Medical Center Utrecht, Utrecht, The Netherlands; 3 Department of Physiology, Amsterdam University Medical Center, location Vrije Universiteit, Amsterdam, The Netherlands; 4 Netherlands Heart Institute, Utrecht, The Netherlands

## Challenges of genetic counselling

In this issue of the Netherlands Heart Journal, two manuscripts and an editorial emphasise the challenges of genetic counselling in cardiomyopathies. Advances in genetic screening have increased the number of genes that can be screened to trace disease-causing variants and enable the identification of a large group of people at risk of developing cardiac arrhythmias and heart failure. The downside, as emphasised in this issue, is that the ability of screening a large panel of genes also uncovers many gene variants of unknown clinical significance (VUS). After the initial enthusiasm about expanded gene panels, scientists became more and more aware that not all known variants are pathogenic and are, in fact, quite common in the general population. The use of the term gene mutation is under debate as mutation implies pathogenicity, while most newly detected gene variants are not pathogenic. Thus, gene variant would be a better term than mutation, and ‘gene variant carrier’ may be used for persons who are at risk of cardiomyopathy. Yet, at the same time, this is where the clinical problem lies. The study by van Lint and colleagues shows that many gene variant carriers may not be at risk of developing a cardiac arrhythmia or cardiac disease [1]. The actual pathogenicity of gene variants appears to vary from disease-causing to being a disease modifier. From a clinical perspective, a new discussion about the clinical utility of large gene panels is warranted, and Dooijes, Siemelink and Baas propose in their editorial that a national consensus about smaller diagnostic gene panels with optimal sensitivity and specificity would aid to optimise genetic screening and counselling, and improve the identification of persons with an actual pathogenic gene variant [2].

## Is there no use at all for large gene panels?

To define the exact role of relatively common genetic variants in Mendelian disorders much more research is required. Broad genetic screening and genome-wide association studies (GWAS) do have relevance for scientific studies to establish whether variants are genetic modifiers, which may be either protective or detrimental. GWAS analysis in a group of patients with Brugada syndrome revealed a cumulative effect of three loci on disease susceptibility, indicating an important modulating role for genetic polymorphisms in cardiac conduction disorders [3]. After the first identification of variants in the gene encoding the giant protein titin as cause of dilated cardiomyopathy [4], follow-up studies indicated that these titin gene variants are less pathogenic when compared with, for instance, lamin variants [5]. Because of its large size, many relatively common gene variants are found in titin. While titin gene variants appear to be less harmful compared with other dilated cardiomyopathy genes, studies in patients carrying titin gene variants showed that increased cardiac stress during an ‘acute’ event such as pregnancy initiates disease [6]. Moreover, other cardiac stress inducers such as chemotherapy and alcohol predispose persons with gene variants to development of disease [7, 8]. Even proven disease-causing variants show a large clinical heterogeneity in families, indicating that the actual onset and progression of cardiomyopathy is the result of the combined effect of gene variants and additional genetic and environmental modifiers. Knowledge of the role of genetic background (polymorphisms) in addition to the pathogenic variant and environmental factors is needed to fully understand the complex and heterogeneous clinical phenotypes, which can range from asymptomatic persons to persons with end-stage heart failure in one family sharing the same pathogenic variant.

## What is needed to define factors that cause cardiomyopathy?

International biobanks and registries with a large number of gene variant carriers are needed to better understand the initiation and progression of genetic heart disease. An example of such an international registry is SHaRe (Sarcomeric Human Cardiomyopathy Registry), which recently reported the results of patients followed in 8 experienced, high-volume hypertrophic cardiomyopathy (HCM) centres. ShaRe showed that patients with sarcomere gene variants of unknown significance were diagnosed earlier and had worse outcomes than HCM patients without gene variants (gene variant-negative), while the risk of adverse events was lower compared with HCM patients with a proven pathogenic gene variant. This study emphasises the need to further study the pathogenicity of newly identified gene variants to improve risk stratification and to enable predictive testing of family members.

Finishing this ‘mission’ will require determination and endurance as genetic heart disease in most individuals is a slowly developing disease. Yet, recent studies highlighted that asymptomatic mutation carriers already show large changes in efficiency of cardiac function in the absence of evident cardiac remodelling (fibrosis, hypertrophy) [9]. We therefore need sensitive in vivo imaging modalities to trace the early pathologic changes in the heart before overt cardiac remodelling. Follow-up studies of both asymptomatic and diseased gene variant carriers over many years are warranted to define the exact pathomechanisms underlying genetic heart disease, which in spite of its frequency is relatively low compared with ischaemic heart disease. Genetic heart disease affects young people and entire families and thereby places a huge burden on society. Ultimately, we may be able to initiate personalised medicine on the basis of the identified gene variant.

## References

[1] van Lint FHM, Mook ORF, Alders M (2019). Large next-generation sequencing panels in genetic heart disease: yield of pathogenic variant and variants of unknown significance. Neth Heart J.

[2] Dooijes D, Siemelink M, Baas AF (2019). Evaluation of gene panels for inherited cardiac disease—is less more?. Neth Heart J.

[3] Bezzina CR, Barc J, Mizusawa Y (2013). Common variants at SCN5A-SCN10A and HEY2 are associated with Brugada syndrome, a rare disease with high risk of sudden cardiac death. Nat Gen.

[4] Herman DS, Lam L, Taylor MR (2012). Truncations of titin causing dilated cardiomyopathy. N Engl J Med.

[5] Jansweijer JA, Nieuwhof K, Russo F (2017). Truncating titin mutations are associated with a mild and treatable form of dilated cardiomyopathy. Eur J Heart Fail.

[6] van Spaendonck-Zwarts KY, Posafalvi A, van den Berg MP (2014). Titin gene mutations are common in families with both peripartum cardiomyopathy and dilated cardiomyopathy. Eur Heart J.

[7] Wasielewski M, van Spaendonck-Zwarts KY, Westerink ND (2014). Potential genetic predisposition for anthracycline-associated cardiomyopathy in families with dilated cardiomyopathy. Open. Heart.

[8] Ware JS, Amor-Salamanca A, Tayal U (2018). Genetic Etiology for Alcohol-Induced Cardiac Toxicity. J Am Coll Cardiol.

[9] Güçlü A, Knaapen P, Harms HJ (2017). Disease stage-dependent changes in cardiac contractile performance and oxygen utilization underlie reduced myocardial efficiency in human inherited hypertrophic Cardiomyopathy. Circ Cardiovasc Imag.

